# Clinical Profile and Outcome of Patients with Acute Kidney Injury Requiring Hemodialysis: Two Years' Experience at a Tertiary Hospital in Rwanda

**DOI:** 10.1155/2018/1716420

**Published:** 2018-03-27

**Authors:** Grace Igiraneza, Benedicte Ndayishimiye, Menelas Nkeshimana, Vincent Dusabejambo, Onyema Ogbuagu

**Affiliations:** ^1^Hemodialysis Unit, Department of Internal Medicine, University Teaching Hospital of Kigali, P.O. Box 655, Kigali, Rwanda; ^2^Department of Internal Medicine, College of Medicine and Health Sciences, University of Rwanda, Rwanda; ^3^Department of Accident & Emergency, University Teaching Hospital of Kigali, P.O. Box 655, Kigali, Rwanda; ^4^Section of Infectious Diseases, Yale University School of Medicine, 135 College Street, New Haven, CT 06520, USA

## Abstract

**Introduction:**

Acute kidney injury (AKI) requiring renal replacement therapy is associated with high mortality. The study assessed the impact of the introduction of hemodialysis (HD) on outcomes of patients with AKI in Rwanda.

**Methods:**

A single center retrospective study that evaluated the clinical profile and survival outcomes of patients with AKI requiring HD [AKI-D] at a tertiary hospital in Rwanda. Data was collected on patients who received HD for AKI from September 2014 to December 2016. Patient demographics, comorbidities, clinical presentation, laboratory tests, and mortality were reviewed and analyzed. Predictors of mortality were assessed using age and gender adjusted multivariate analyses.

**Results:**

Of the 82 eligible patients, median age was 38 years (IQR 28–57 years). Males comprised 51% of the cohort. Infectious diseases including malaria, pneumonia, and sepsis (35.1%) and pregnancy-related conditions (26.9%) were the most frequent comorbidities. Pulmonary oedema (54.9%) and uremic encephalopathy (50%) were top indications for HD. Mortality was 34.1%. On multivariate analysis, receipt of <5 sessions of HD (OR = 4.01, 95% CI 1.185–13.61, *P* = 0.026) and hyperkalemia (OR = 3.23, 95% CI 1.040–10.065, *P* = 0.043) were associated with mortality.

**Conclusion:**

The availability of acute hemodialysis in Rwanda has resulted in improved patient survival and persistent hyperkalemia predicted higher mortality.

## 1. Introduction

Acute kidney injury (AKI) is a common medical problem among hospitalized patients and may be associated with multiple etiologies, occurring singly or in combination, including infectious diseases or conditions such as diarrheal disease, HIV, malaria, glomerulonephritis and sepsis, toxins or herbal medications, autoimmune diseases, pregnancy-related conditions, trauma-related tubular injury, and iatrogenic causes including medications such as nonsteroidal anti-inflammatory drugs, hypovolemia, and contrast induced nephropathy [1, 2]. While there is limited data, the incidence of AKI among hospitalized patients in Africa is estimated at 0.3–1.9% [3]. Country specific studies such as those in Malawi found an incidence of AKI of 17.2% [4]. Mortality rates among hospitalized patients with AKI may be as high as 44.4% [4].

Renal replacement therapy (RRT), though rudimentary at the time, became available in the early 1950s [5]; however, even in the current decade, the lack of capacity and infrastructure for renal replacement therapy continues to plague resource-limited settings, such that AKI requiring HD is a lethal condition. Even where services are available, limited access to facilities where they exist and their prohibitive cost are obstacles to patients utilizing the intervention. Hemodialysis was introduced in Rwanda at a single health facility a decade ago with limited machines and renewables such that its accessibility was quite problematic by patients who needed it. Further limiting access was that the procedure was not covered by the community-based health insurance (CBHI),* Mutuelle de Sante, *which is the health insurance provider for more than 80% of the Rwandan population [6].

In 2014, the Rwandan government in response to concerns about high mortality related to lack of RRT access expanded the availability of HD equipment to more healthcare facilities including the University Teaching Hospital of Kigali. Also there was an expansion of coverage of medical procedures to include 6 weeks of acute HD for patients with AKI for those utilizing the government-run community-based health insurance scheme. Currently there are an estimated 20 dialysis machines in 3 referral hospitals that provide access to more patients. However, the impact of the expanded availability of RRT has not been assessed. This is the first study to describe the survival outcomes and predictors of mortality among patients with acute kidney injury requiring renal replacement therapy in Rwanda.

## 2. Materials and Methods

This was a retrospective study conducted at the hemodialysis unit of the Kigali University Teaching of Kigali (CHUK). CHUK is a 524-bed, government-run tertiary level hospital that is located in Kigali, the capital city of Rwanda, and is the largest public hospital receiving patients referred from all parts of the country who need specialized medical and surgical services.

Patients who had hemodialysis from September 2014 to December 2016 were identified from the hospital's hemodialysis registry. Eligible patients were those with a diagnosis of AKI (per Kidney Disease Improving Global Outcomes [KDIGO] criteria and/or provider definition) prior to initiation of HD. Individuals who had a diagnosis of end-stage renal disease (ESRD) at the initiation of HD, patients below 16 years of age, and those with incomplete or missing medical records were excluded.

Hemodialysis was typically performed for 4-hour sessions thrice weekly except for patients with uremic encephalopathy, for whom initial HD sessions were conducted for only 2 hours to prevent disequilibrium syndrome. Double lumen catheters are utilized at our facility for vascular access for HD and placed in internal jugular or femoral veins. Blood flow and dialysate flow rates typically range within 250–300 ml/min and 500–600 ml/min, respectively. Ultrafiltration volume was dependent on patient's hemodynamic status. As continuous renal replacement therapy is not available at our facility, patients that were hemodynamically unstable did not receive HD.

Patients' demographics (age, sex, and residential address) and clinical data at the time of initiation of HD such as hospital wards, vital signs, and comorbidities were obtained from patients' medical charts. Subsequently, number of hemodialysis sessions received and the total days of hospital stay were obtained from patients' medical records. Hypertension was defined as a systolic blood pressure equal to or greater than 140 mmHg and/or a diastolic pressure equal to or greater that 90 mmHg, while hypotension was defined as a systolic blood pressure of 90 mmHg or less and or diastolic blood pressure of 60 mmHg and below. Stages of hypertension were graded as follows: mild hypertension: 140/90–159/99 mmHg; moderate hypertension: 160/100–179/109 mmHg; and severe hypertension: >180/110 mmHg.

Predialysis laboratory tests results were recorded from either patients' charts or hospital electronic laboratory database. Hyperkalemia was defined as serum potassium greater than 5.5 mmol/L. Mild and severe hyperkalemia were defined as 5.6–6.59 mmol/L and above 6.6 mmol/L, respectively. Indications for hemodialysis including pulmonary edema, hyperkalemia, and uremic encephalopathy were recorded as documented by providers. Patients' mortality outcome at the end of hospital stay was also recorded. Data was captured in an electronic database using Microsoft Excel.

Statistical analyses were performed using Stata Software 13.0. Continuous variables were reported as means with standard deviation (SD) or medians with interquartile range (IQR). Underlying comorbid conditions of AKI patients and in-hospital clinical outcome of patients that sustained AKI and received hemodialysis were reported as simple frequencies. Bivariate analysis was performed to assess the relationship between study variables (including patient demographics, comorbidities present, indications for hemodialysis, and length of hospital stay) and mortality. Factors found to be significantly associated with mortality on bivariate analysis (*P* < 0.05) were analyzed with a multivariate model to determine the predictors of in-hospital mortality using logistic regression. Odds ratios were calculated after adjusting for sex and gender. *P* values less than 0.05 were considered statistically significant.

## 3. Results

### 3.1. Subject Eligibility and Baseline Demographics

**Figure 1:**
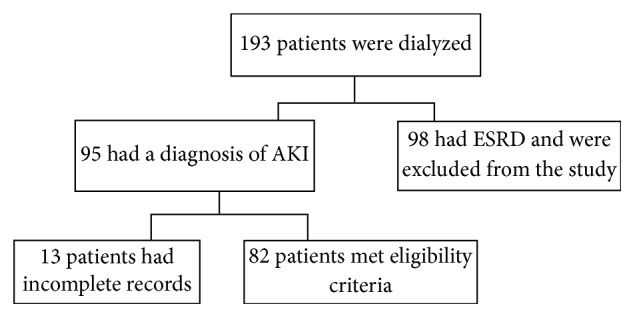
Screening process and eligibility. AKI: acute kidney injury; ESRD: end-stage renal disease.

Over the study period, there were 193 patients with renal failure requiring RRT. Ninety-five (95) patients had AKI. Of those who were dialyzed for AKI, 13 patients had incomplete medical records and were excluded from the study (see Figure 1). Of the 82 eligible patients, males accounted for 51.2%. The median age was 38 years (IQR 28–57). The mean length of hospital stay was 23.3 days (SD +/− 14). The majority of the study participants (62.2%) were admitted on medical wards (see Table 1).

### 3.2. Clinical Characteristics of Cohort

**Figure 2:**
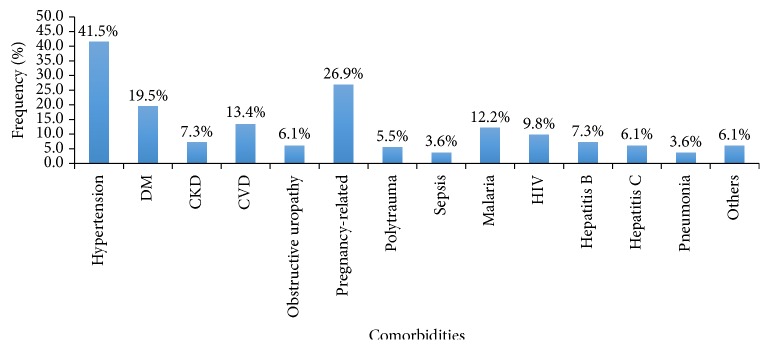
Comorbidities in patients that sustained AKI. DM: diabetes mellitus; CKD: chronic kidney disease; CVD: cardiovascular diseases; HIV: human immunodeficiency virus; “Others” category includes cancers and use of herbal medications.

Severe hypertension was observed in 23.2% of the cohort and hyperkalemia >6.6 mmol/L in 40.2% (Table 1). Medical comorbidities present in the cohort included infectious diseases and conditions such malaria (12.1%), pneumonia (3.7%), and sepsis (3.7%). Noncommunicable diseases such as hypertension (41.5%), diabetes (20.4%), and CKD (7.3%) were also frequently present. Polytrauma accounted for 5.5% while pregnancy-related conditions accounted for 26.9%. The latter included eclampsia, postpartum hemorrhage, post-cesarian section peritonitis (see Figure 2).

### 3.3. Timing and Indications for HD

**Figure 3:**
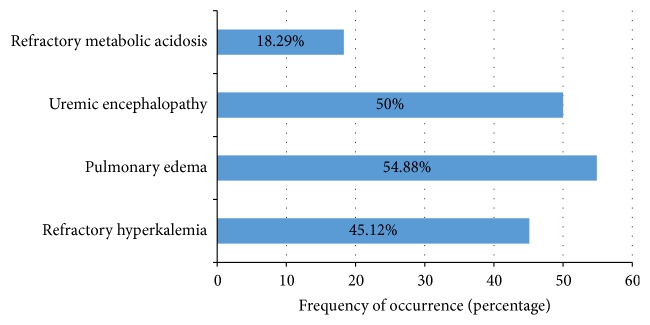
Indications for hemodialysis.

For more than half of the patients (57.3%), hemodialysis was initiated within three days of their hospital stay. The number of dialysis sessions per patient ranged from one to sixteen with an average of 6.5 sessions per patient. The most common indications for dialysis were fluid overload primarily manifesting as pulmonary oedema (54.8%), followed by uremic encephalopathy (50%) and refractory hyperkalemia (45.1%) (see Figure 3). Some patients had multiple indications for urgent dialysis.

### 3.4. Clinical Outcome and Predictors of AKI Mortality

With regard to the clinical outcome, 65.9% survived and were discharged home. On multivariate analysis, after adjusting for sex and gender, receipt of <5 sessions of HD (OR = 4.01, 95% CI 1.185–13.611, *P* = 0.026) and hyperkalemia were associated with not surviving (OR = 3.23, 95% CI 1.040–10.065, *P* = 0.043) (see Table 2).

## 4. Discussion

Multiple studies have shown that AKI occurs frequently among hospitalized patients and contributes significantly to increased morbidity and mortality, prolonged hospital stay, and healthcare costs including increased needs for critical care [7–9]. Understanding the proximate causes of AKI and potentially modifiable etiologies continues to be the focus of research [10].

Our study showed that acute kidney injury requiring hemodialysis (AKI-D) primarily affected young adults (median age 38 years), a finding that is unlike reports from Western countries where the most frequently affected people are the elderly [11]. On the other hand, studies from other sub-Saharan African countries, similar to our findings, show that AKI impacts younger individuals with mean age of occurrence of AKI being 37 and 44 years in Uganda and South Africa, respectively [12, 13]. The young age of AKI-D patients in this study may be simply reflective of the demographics of the Rwandan population where most individuals are below the age of 50 years and people above 65 years constitute only 3% of the Rwandan population according to the Rwandan 4th Population and Housing Census of 2012 [14], but it may also reflect the underlying causes of AKI, particularly infections and pregnancy-related conditions which impact younger individuals. The finding that young and middle aged people constitute the majority of cases of AKI is concerning as it has downstream socioeconomic costs including decreased productivity and increased economic burden to their households and society [15]. However, as patients aged 65 and above accounted for 9.76% of cases in this cohort, compared to overall patient demographics, they were also disproportionately impacted by AKI. This is consistent with older age being a known risk factor for AKI.

Our study found that acute infections were common comorbidities observed in AKI-D patients including malaria (12.2%), pneumonia (3.6%), and sepsis (3.6%). The World Health Organization (WHO) estimates that malaria may result in AKI in 1% of cases [16], but can be as high as 4% in some malaria endemic regions, being more prevalent in adults and older children [17, 18]. The link between these infectious diseases and AKI is well described; for example, AKI in malaria may be multifactorial resulting from insensible fluid losses from fever, gastrointestinal fluid loss secondary to vomiting, direct parasite invasion of tubular cells, renal hypoperfusion secondary to systemic vasodilatation, toxicity from hemolysis products, or direct kidney injury from malaria treatment. Fortunately, malaria-related AKI is usually reversible if patients survive malaria [16]. In spite of 82% of Rwandan households owning at least one long lasting insecticide treated mosquito net [19], 1.9 million malaria cases occurred in 2015. This high number of incident cases might explain the relatively disproportionate contribution of malaria to AKI in this study (12.2%). However, it may also reflect a selection bias as the study took place at a referral hospital in Rwanda where all complicated malaria cases are sent. Sepsis related AKI is common in developing countries and may be attributable to multiple factors including late onset of presentation with infection, poor adherence to treatment protocols, adverse effects of treatments offered, and limited critical care capacity.

Consistent with other reports from Africa, our study also found that, in addition to infectious diseases, pregnancy-related conditions are frequently associated with severe renal injury requiring renal replacement therapy [2]. Pregnancy-related AKI is one of top causes of mortality among young women in low and middle income countries, while the condition is uncommon in the developed world [2]. These include conditions such as post-caesarian section peritonitis, post- and antepartum hemorrhage, septic abortion, preeclampsia, and eclampsia. In alignment with our findings, previous studies on AKI in sub-Saharan Africa have reported obstetric complications as an important cause of AKI [2, 11]. In South Africa, pregnancy-related disorders, most notably septic abortion and preeclampsia, were responsible for 15% of AKI requiring dialysis [20]. These conditions are frequently a measure of the quality of obstetric care in the locations where they occur, and thereby improving access to and promoting services as appropriate may prevent some of these events.

Worldwide, AKI-related mortality is estimated at 21% and can be as high as 45% in stage 3 AKI patients [21]. The mortality rate we observed for AKI-D patients of 34.1% is high but without the recently introduced HD services in the country, this rate could have approximated 100%. This clearly supports the life-saving impact of HD services. On multivariate analysis, we found that hyperkalemia was a predictor of mortality along with receipt of <5 sessions of HD. While we did not specifically assess causality of mortality, it is plausible that uncontrolled hyperkalemia was responsible for many deaths. This may be attributable to poor laboratory services to allow for frequent electrolyte monitoring and may reflect late referral to our facility and unavailability of medications such as oral binding resins which can have a durable effect on reducing potassium levels. The finding that patients who died had less sessions of HD probable reflects AKI severity and/or absence of renal recovery referable to the underlying etiologic disease.

Our study had several limitations. Being a retrospective study, quality of data was dependent on accuracy of documentation by patient care providers. As the decision for HD was made by individual clinicians, the patients in our study may not be reflective of all patients with AKI in our facility as such decisions typically include considerations of cost, insurance status, and perceived prognosis and benefit of RRT. Furthermore, the etiologies and associated conditions in patients with AKI requiring HD may be different from those who do not require RRT. Our patients, seen at a tertiary facility where more severe cases are referred, may not be representative of those at lower level health facilities within Rwanda. We did not assess specific causes of mortality especially those that may be attributable to renal versus nonrenal conditions. Also, patients who were ineligible for HD including those who were hemodynamically unstable to tolerate HD (who may have benefitted from other modalities of HD such as continuous renal replacement therapy which, unfortunately, is not available in our center) were not included in the study such that mortality reported may be an underestimation of mortality among patients with severe AKI with an indication for HD.

## 5. Conclusion

Our study showed that AKI-D occurred principally among middle aged adults with infectious diseases and pregnancy-related conditions being the most common comorbidities. AKI-D carries a high mortality rate in Rwanda although improved where RRT is available. More efforts should be focused on AKI prevention including improved treatment of underlying conditions, timely diagnosis and complication management, and expanding access to RRT including HD before fatality occurs.

## Figures and Tables

**Table 1:** Selected demographics, clinical characteristics, and laboratory parameters of study participants.

Variable	Frequency or median (*N* = 82)	Percent (%) or IQR
*Sex*		
Male	42	51.2
*Age*		
25 and younger	17	20.8
26–35	20	24.3
36–45	12	14.7
46–64	25	30.4
65 and above	8	9.8
*Ward*		
Medical	51	62.2
OB-GYN	22	26.9
Surgery	7	8.5
ICU	1	1.2
A&E	1	1.2
*Vital signs*		
*Blood pressure*		
Hypotension	4	4.9
Normal	30	36.6
Mild HTN	18	21.9
Moderate HTN	11	13.4
Severe HTN	19	23.2
*Laboratory tests *		
*Serum potassium*		
Mild hyperkalemia	10	12.2
Severe hyperkalemia	33	40.2
* Serum creatinine (mg/dl)*	10.1	6.2–15.7
* Serum urea (mmol/L)*	33	21–50

Hypotension: BP < 90/60 mmHg; normal BP: 91/61–139/89 mmHg; mild hypertension: 140/90–159/99 mmHg; moderate hypertension: 160/100–179/109 mmHg; severe hypertension: >180/110 mmHg; mild hyperkalemia: 5.6–6.59 mmol/L; severe hyperkalemia: above 6.6 mmol/L; *N*: number of subjects; IQR: interquartile range; OB-GYN: obstetrics and gynecology; ICU: intensive care unit; A&E: accident and emergency; HTN: hypertension.

**Table 2:** Predictors of mortality in patients with acute kidney injury requiring hemodialysis.

Variable	Bivariate			Multivariate		
	Survivors	Nonsurvivors	*P* value	Odds ratio	95% CI	*P* value
Age						
</=45	34 (%)	15 (%)	0.411			
>45 years	20	13				
Sex						
Male	29	13	0.532			
Female	25	15				
HIV						
No	51	23	0.115			
Yes	3	5				
HD sessions						
<5	3	12	<0.0001	4.01	1.185–13.611	0.026
>6	51	8				
Hyperkalemia						
No	34	11	<0.0001	3.23	1.040–10.065	0.043
Yes	20	17				
Acidosis						
No	45	22	0.597			
Yes	9	6				
Pulmonary edema						
No	23	14	0.523			
Yes	31	14				
Hypertension						
No	33	15	0.511			
Yes	21	13				
CKD						
No	49	27	0.659			
Yes	5	1				
CVD						
No	48	23	0.395			
Yes	6	5				

CI: confidence interval; HIV: human immunodeficiency virus; HD: haemodialysis; CKD: chronic kidney disease; CVD: cardiovascular diseases.
